# Estimating SARS‐CoV‐2 transmission in educational settings: A retrospective cohort study

**DOI:** 10.1111/irv.13049

**Published:** 2022-09-20

**Authors:** Mattia Manica, Piero Poletti, Silvia Deandrea, Giansanto Mosconi, Cinzia Ancarani, Silvia Lodola, Giorgio Guzzetta, Valeria d'Andrea, Valentina Marziano, Agnese Zardini, Filippo Trentini, Anna Odone, Marcello Tirani, Marco Ajelli, Stefano Merler

**Affiliations:** ^1^ Center for Health Emergencies, Bruno Kessler Foundation Trento Italy; ^2^ Epilab‐JRU, FEM‐FBK Joint Research Unit Trento Italy; ^3^ Prevention Department Agency for Health Protection Pavia Italy; ^4^ Department of Public Health, Experimental and Forensic Medicine University of Pavia Pavia Italy; ^5^ Dondena Centre for Research on Social Dynamics and Public Policy, and CovidCrisisLab Bocconi University Milan Italy; ^6^ Directorate General for Health, Lombardy Region Milan Italy; ^7^ Laboratory for Computational Epidemiology and Public Health, Department of Epidemiology and Biostatistics Indiana University School of Public Health Bloomington Indiana USA

**Keywords:** contact tracing, COVID‐19, school transmission

## Abstract

**Background:**

School closures and distance learning have been extensively adopted to counter the COVID‐19 pandemic. However, the contribution of school transmission to the spread of SARS‐CoV‐2 remains poorly quantified.

**Methods:**

We analyzed transmission patterns associated with 976 SARS‐CoV‐2 exposure events, involving 460 positive individuals, as identified in early 2021 through routine surveillance and an extensive screening conducted on students, school personnel, and their household members in a small Italian municipality. In addition to population screenings and contact‐tracing operations, reactive closures of class and schools were implemented.

**Results:**

From the analysis of 152 clear infection episodes and 584 exposure events identified by epidemiological investigations, we estimated that approximately 50%, 21%, and 29% of SARS‐CoV‐2 transmission was associated with household, school, and community contacts, respectively. We found substantial transmission heterogeneities, with 20% positive individuals causing 75% to 80% of ascertained infection episodes. A higher proportion of infected individuals causing onward transmission was found among students (46.2% vs. 25%, on average), who also caused a markedly higher number of secondary cases (mean: 1.03 vs. 0.35). By reconstructing likely transmission chains from the entire set of exposures identified during contact‐tracing operations, we found that clusters originated from students or school personnel were associated with a larger average cluster size (3.32 vs. 1.15) and a larger average number of generations in the transmission chain (1.56 vs. 1.17).

**Conclusions:**

Uncontrolled SARS‐CoV‐2 transmission at school could disrupt the regular conduct of teaching activities, likely seeding the transmission into other settings, and increasing the burden on contact‐tracing operations.

## 1 BACKGROUND

School closures and the replacement of in‐person school attendance with distance learning were extensively implemented during the first 2 years of the COVID‐19 pandemic. Such policies have been associated with a heated public debate due to their impact on the quality of students' education, the costs and resources required to provide safe educational environments, and the well‐being of children and their parents.^1^, ^2^ Compared with adults, children are less likely to develop symptoms and thus of being reported to surveillance systems.^2^, ^3^, ^4^ However, evidence of viral circulation in schools has been repeatedly found.^2^, ^5^, ^6^, ^7^, ^8^, ^9^ School‐related disease incidence was found to increase with the proportion of students receiving in‐person education,^10^ while, at the same time, infection prevalence among students and teachers was associated with COVID‐19 incidence in the community.^11^, ^12^ Most published studies are based on the analysis of epidemiological trends observed after schools' reopening and therefore at risk of confounding and collinearity from the simultaneous release of other non‐pharmacological interventions.^13^ The quantification of the contribution of school to the overall SARS‐CoV‐2 transmission thus remains elusive.

In this study, we analyze 460 SARS‐CoV‐2 positive individuals and 976 contacts identified by routine surveillance and through an extensive screening of the student population and of their households during an outbreak in Mede, Italy, in early 2021. The analysis of exposures and infection episodes identified by epidemiological investigations allowed us to provide quantitative estimates of the role of students in the spread of the infection, considering potential heterogeneities in the risk of infection and onward transmission after the exposure to SARS‐CoV‐2 in households, schools, and the community.

## 2 METHODS

### 2.1 Study population

In February 2021, a rapid upsurge of symptomatic cases was detected in Mede, a small municipality of the Lombardy region, Italy (6326 inhabitants). A non‐negligible set of positive cases were identified among students, raising concern about widespread circulation of SARS‐CoV‐2 in schools. The outbreak involved a crèche, a kindergarten, and the local main school, which consists of a primary (259 pupils) and a middle school (155 students). On February 5, 2021, all students of three classes (two from the kindergarten and one from the primary school) were quarantined at home according to protocols implemented in the country to prevent school outbreaks.^14^ The progressive increase of cases determined the closure of the kindergarten on February 7; the closure of the crèche, the primary, and the secondary school on February 16; and the application of the highest level of restrictions to the whole municipality on February 17. Between February 17 and March 23, local health authorities carried out a free screening campaign based on PCR tests and involving all individuals connected to the schools (i.e., students/school personnel and their household members). Information about the household of screened individuals, the class, and the school attended by tested students was recorded. The screening was followed by tracing and testing of contacts of identified SARS‐CoV‐2 positive individuals. Epidemiological investigations were conducted by public health authorities to identify likely infection episodes between involved individuals. The definitions of COVID‐19 cases and case contacts adopted in Italy can be found in Poletti et al.^3^

**FIGURE 1:**
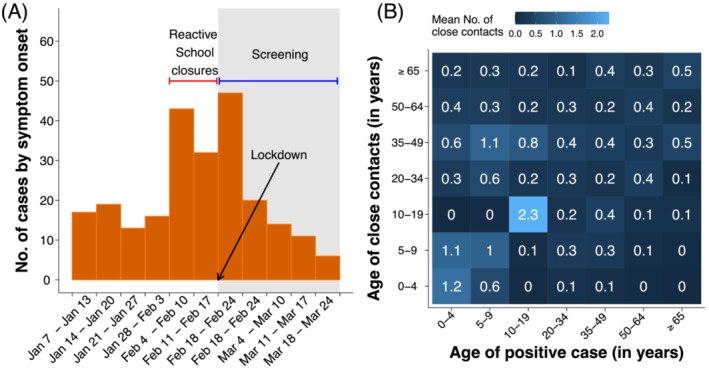
(A) Time series of symptomatic cases reported in the municipality of Mede by week of symptom onset. (B) Contact matrix representing the average number of close contacts reported by positive cases

The analyzed sample consists of all SARS‐CoV‐2 positive individuals and their contacts identified between January 7 (the date of school reopening after the Christmas holidays) and March 10, 2021 (i.e., 3 weeks after the strengthening of restrictions in the whole municipality). Positive individuals were classified as symptomatic infections if they showed upper or lower respiratory tract symptoms or fever ≥37.5°C.^3^ Respiratory symptoms included dry cough, dyspnea, tachypnea, difficulty breathing, shortness of breath, sore throat, and chest pain or pressure.^3^ Figure 1A shows the time series of symptomatic cases identified in Mede during the study period, along with the timeline of interventions implemented in the municipality.

### 2.2 Exposures, epidemiological links, and the chance of onward transmission

Potential infection episodes were identified by collating the set of possible infectors for each positive individual, based on the close contacts reported by each ascertained infection during epidemiological investigations. Based on these epidemiological links, we estimate the number and age of secondary cases caused across different settings by infectors of different ages. We discarded positive individuals reporting more than one potential infector and infector–contact pairs for which was not possible to establish who was the primary infector and who was the secondary case.

To define the likely source of infection, we assumed that exposures occurring between cohabiting individuals took place in their household. Exposures recorded between individuals attending the same school (either as students or school personnel) but not sharing the same household were considered as school exposures. Exposures outside the household and school were classified as occurred in the community. Positive cases without a history of exposure to SARS‐CoV‐2 were assumed to be associated to an unknown source of infection in the community.

To check for heterogeneity in transmission, we fit a negative binomial distribution to the offspring distribution estimated by calculating the number of secondary cases caused by each positive individual.

We explored the association between exposures and PCR positive results to SARS‐CoV‐2 infection, using a generalized linear mixed‐effects model (GLMM) with logit link. The observational unit was defined as the exposure of close contacts to an identified SARS‐CoV‐2 positive case. The response variable was the PCR test result of the contact after the exposure. Model covariates included the gender and age of both the contact and their potential infector as well as the setting of exposure (i.e., within household, within school, or in the community). Statistical significance of the parameters of the logistic regression was assessed through the Wald test. Exposures of female contacts to a positive female in the household were considered as the reference group.

We carried out an additional analysis considering all potential exposures (i.e., including individuals with multiple exposures) ascertained during contact‐tracing activities. To deal with multiple exposures, we randomly sampled the potential infector among the pool of positive contacts. Specifically, infectors were assigned with equal prior probability between possible links while checking for consistence in the resulting transmission chain (i.e., rejecting circular transmission within the analyzed clusters, see Figure S1). The procedure was repeated 1000 times to obtain different instances of consistent transmission chains. Each simulated instance was analyzed in terms of (i) the number and age of secondary cases caused across different settings by infectors of different ages; (ii) the size of identified clusters of infection, defined as separate sets of two or more SARS‐CoV‐2 positive individuals having an epidemiological link; and (iii) the length of different transmission chains, defined as the number of generations within clusters. Obtained results are presented in terms of mean values and 95% prediction intervals (PI) computed over the 1000 instances of the transmission chains.

## 3 RESULTS

### 3.1 Descriptive statistics

We collated a dataset of 822 individuals tested for SARS‐CoV‐2 infection between January 7 and March 10, 2021, representing 13% of the residents of the focus municipality (Mede). The median age of the tested individuals was 36 years (IQR: 11–53, ranging from 1 month to 98 years); 52.3% individuals were female. Out of the 822 tested individuals, 460 resulted PCR positive for SARS‐CoV‐2 infection, including 237 (51.5%) showing symptoms while 183 (39.8%) were asymptomatic (for 40 positive individuals, 8.7%, this information was not reported). The median age of symptomatic and asymptomatic cases was 43 years (IQR: 28–56) and 31 years (IQR: 10–49), respectively. Among the ascertained infections, 311 were identified through standard surveillance and 149 through the scholastic investigation. The positivity ratio in these two groups of tested individuals was 66.9% (311/465) and 41.7% (149/357), respectively. The corresponding symptomatic ratio was 57.9% (180/311) and 38.3% (57/149). Overall, 203 (24.7%) tested individuals were students and 13 (1.6%) were school personnel. Out of the 460 ascertained infections, 82 (17.8%) were students and 8 (1.7%) were school personnel. A detailed description of the analyzed sample is reported in Table 1.

**TABLE 1:** Description of the analyzed sample

			Tested	SARS‐CoV‐2 positive (%)	Symptomatic cases (%)
Overall	Overall		822	460 (56%)	237 (51.5%)
	Age class (years)	0–5	55	17 (30.9%)	7 (41.2%)
		6–10	84	42 (50%)	9 (21.4%)
		11–20	153	68 (44.4%)	30 (44.1%)
		21–35	104	69 (66.3%)	34 (49.3%)
		36–50	191	120 (62.8%)	75 (62.5%)
		51–65	108	63 (58.3%)	42 (66.7%)
		Above 66	127	81 (63.8%)	40 (49.4%)
	Sex	Female	430	257 (59.8%)	140 (54.5%)
		Male	392	201 (51.3%)	97 (48.3%)
Scholastic screening	Overall		357	149 (41.7%)	57 (38.3%)
	Age class (years)	0–5	38	10 (26.3%)	5 (50%)
		6–10	57	25 (43.9%)	4 (16%)
		11–20	90	30 (33.3%)	13 (43.3%)
		21–35	44	28 (63.6%)	9 (32.1%)
		36–50	68	30 (44.1%)	17 (56.7%)
		51–65	29	11 (37.9%)	7 (63.6%)
		Above 66	31	15 (48.4%)	2 (13.3%)
	Sex	Female	186	89 (47.8%)	36 (40.4%)
		Male	171	60 (35.1%)	21 (35%)
Routine surveillance	Overall		465	311 (66.9%)	180 (57.9%)
	Age class (years)	0–5	17	7 (41.2%)	2 (28.6%)
		6–10	27	17 (63%)	5 (29.4%)
		11–20	63	38 (60.3%)	17 (44.7%)
		21–35	60	41 (68.3%)	25 (61%)
		36–50	123	90 (73.2%)	58 (64.4%)
		51–65	79	52 (65.8%)	35 (67.3%)
		Above 66	96	66 (68.8%)	38 (57.6%)
	Sex	Female	244	168 (68.9%)	104 (61.9%)
		Male	221	143 (64.7%)	76 (53.1%)

The average number of close contacts reported by positive individuals with household members was 1.1 (IQR: 0–2); 0.6 (IQR: 0–1) contacts per person were identified in the community. Positive students reported an additional 3.2 (IQR: 0–2) contacts with schoolmates or school personnel, resulting in a higher average number of close contacts experienced overall (5.0 vs. 1.5 for non‐students; Wald test p value < 0.0001). The average number of contacts that an individual of a given age has with individuals in other age groups can be visualized in the form of a matrix (Figure 1B). The obtained contact matrix shows that the highest contact rate was reported by individuals aged 10–20 years with individuals in the same age group.

We identified 976 potential exposure events: 432 consisted of either single or multiple negative exposure events, 152 led to the identification of a clear infection episode, 326 were associated with positive individuals reporting contacts with multiple positive individuals, and 66 were associated with positive individuals with an unknown source of infection.

### 3.2 SARS‐CoV‐2 transmission patterns

**FIGURE 2:**
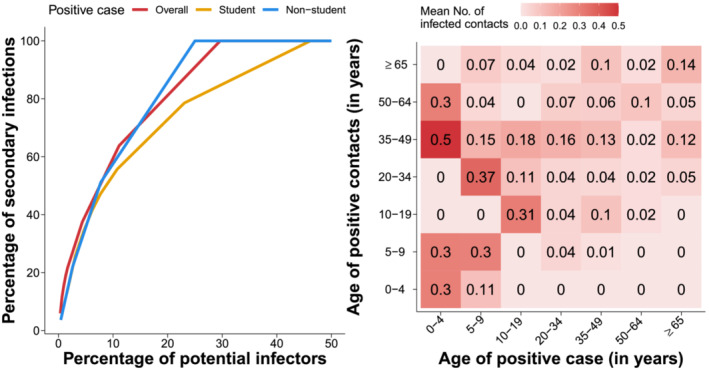
(A) Distribution of secondary infections generated by identified positive cases. (B) Transmission matrix representing the average number of infections caused in each age group by positive cases of different ages

By removing cases and case contacts associated with unclear infection episodes or exposures, we analyzed 297 ascertained SARS‐CoV‐2 infections and 584 exposure events. We found that 209 positive individuals (70.4% of potential infectors, 95%CI 64.8% to 75.5%) did not cause any secondary infection. The average number of secondary cases caused by a positive individual was estimated to be 0.51 (range: 0–9)—we stress that the average number of secondary infections must be lower than 1 in any contained outbreak.^15^ However, a higher proportion of individuals causing onward transmission was found among positive students (46.2% 95%CI 33.7–59.0 vs. 25.0% 95%CI 19.6–31.1), who were also found to cause on average a markedly higher number of secondary cases (1.03, range: 0–9 vs. 0.35, range: 0–3). The number of secondary infections caused by school personnel and by individuals unrelated with the school setting was 0.40 and 0.35, respectively. We estimated the distribution of the number of secondary infections to follow a negative binomial distribution with overdispersion (shape parameter) 0.48 (bootstrapped 95%CI 0.30–0.75), implying that 20% of positive cases were responsible for 75% to 80% of all secondary cases (Figure 2A). A similar heterogeneity in the transmission was found among students (overdispersion: 0.55, bootstrapped 95%CI 0.31–1.28).

We estimated that 76 infection episodes were linked to a household contact (out of the 249 analyzed household exposures), 32 infections episodes were linked to a scholastic contact (out of 165 exposures); 44 infections occurred in the community (out of 170 exposures). Accordingly, infection episodes represented 30.5% (95%CI 24.9–36.7), 19.4% (95%CI 13.7–26.3), and 25.9% (95%CI 19.5–33.1) of exposures occurred in the household, school, and community, respectively. However, results obtained through a generalized linear model showed that, after adjusting for possible confounders such as the contacts' age, the risk of infection was not statistically different for exposures occurred in different settings (household, school, and community; see Table S1).

Based on the identified infection episodes, we reconstructed an age‐specific matrix representing the average number of infections caused in each age group by a positive case, stratified by the age of the infector (Figure 2B). The highest transmission intensity was found from young children to adults and between children of similar age (possibly reflecting contacts between siblings or schoolmates). The average number of secondary cases caused by any positive individual at home and in the community was 0.30 (range: 0–3) and 0.20 (range: 0–3), respectively. Positive students caused an additional 0.52 (range: 0–9) cases among school‐related contacts (schoolmates or school personnel).

**FIGURE 3:**
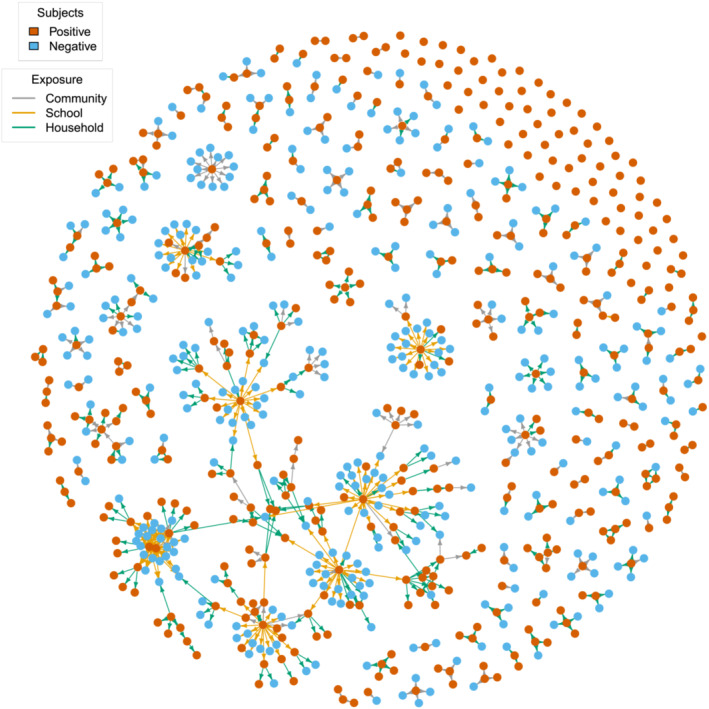
Contact networks representing all exposure events identified by epidemiological investigations. The color of the nodes represents the infectious status of each tested individual. Subjects who experienced both negative and positive exposures (namely, five individuals) are represented twice. Edges represent the exposure event between two subjects, therefore connecting a positive case to his/her close contacts. The color of the edges represents the setting of exposure.

Similar results were obtained by including in the analysis also positive individuals with multiple exposures (see Figure 3 and Appendix S1). In this case, the analysis of potential transmission chains led us to identify 144 (95%PI: 140–148) clusters of infections associated with an average number of 4.49 (95%PI: 4.35–5.64) close contacts up to a maximum of 81.1 (95%PI: 76–87). Most of the identified clusters (121, 95%PI: 117–125) originated from individuals not related with the scholastic setting. However, clusters originating from students or school personnel (23 clusters 95%PI: 21–25) showed a larger number of generations in the transmission chain (1.56, 95%PI: 1.42–1.70 vs. 1.17, 95%PI: 1.13–1.21), a larger number of infection episodes per cluster (3.32, 95%PI: 2.79–3.83 vs. 1.15, 95%PI: 1.06–1.24), and a larger set of associated close contacts (11.3, 95%PI: 10.3–12.9 vs. 3.15, 95%PI: 2.95–3.35).

## 4 DISCUSSION AND CONCLUSIONS

In this work, we analyzed a scholastic outbreak in an Italian municipality. The collected records included PCR positive individuals and their close contacts identified by routine surveillance and through an extensive screening conducted on students, school personnel, and their household members.

Despite protocols in place to curb SARS‐CoV‐2 transmission during in‐person school attendance, our analysis suggests that younger age groups were deeply involved in the spread of the infection. The average number of secondary cases caused by an infected student was significantly larger as compared with other individuals (1.03 vs. 0.35). This result well compares with evidence from France suggesting that, in spring 2021, the school‐specific reproductive number was significantly higher than that estimated for the community.^16^ About 21.1% of the identified infection episodes occurred because of interactions between schoolmates. The transmission between schoolmates was associated with longer transmission chains, a larger number of individuals were exposed to the infection (on average 5.0 contacts were named by positive students as compared with 1.5 for other individuals), and a larger number of infections were estimated for cluster originated from school‐related exposures. This implies that uncontrolled transmission in the student population could disrupt the regular conduct of teaching activities and leading to a harsh burden for contact‐tracing operations at the same time. In addition, the circulation of SARS‐CoV‐2 in the scholastic population may entail a high risk of importation of the infection into a large set of households from where it can reach age segments at higher risk of infection and disease.^3^, ^5^

In line with previous studies,^16^, ^17^, ^18^, ^19^ the estimated distribution of secondary infections indicates a substantial transmission heterogeneity, with 20% of positive individuals causing 75% to 80% of all transmission events. This heterogeneity suggests that control programs targeting contexts responsible for most of the transmission could be effective in limiting the spread of the infection, including in educational settings.^16^, ^17^, ^20^

A key limitation of our study is that we were not able to collect the time of exposure(s) for positive individuals. On one hand, this prevented us to apply standard Bayesian approaches to reconstructing the occurred transmission chains.^9^, ^21^ On the other hand, when an infection episode was identified between individuals involved in the scholastic settings, we cannot exclude that the transmission occurred outside school. As for other epidemiological investigations, it is likely that some contacts were not identified or remained untested. As testing and screening efforts across different settings were unbalanced, biases in the quantification of contribution of different ages in the transmission cannot be excluded. Finally, data presented here refer to the first months of 2021, when the Alpha variant was emerging in Italy and the vaccination rate was sharply increasing. However, the impact of vaccination during the study period should be negligible (less than 16% of the population was vaccinated with 1 dose by the end of the study period^22^). Finally, the analyzed data do not provide sufficient granularity to provide estimates of school transmission vs. transmission between schoolmates, which may potentially occur in other social settings.

The contribution of students to SARS‐CoV‐2 transmission and the high prevalence in the student population we have found through an extensive screening hint at the difficulties in tracking asymptomatic infections and the challenges implementing reactive class/school closures to interrupt SARS‐CoV‐2 transmission.^12^, ^16^, ^23^, ^24^ The persistent circulation of SARS‐CoV‐2 and the shift of the age of cases towards younger ages observed throughout Europe in 2022 highlight the need of closely monitoring the epidemiological situation in schools.

## CONFLICT OF INTEREST

MA has received research funding from Seqirus. The funding is not related to COVID‐19. All other authors declare no competing interest.

## ETHICS APPROVAL AND CONSENT TO PARTICIPATE

Data collection and analysis were part of outbreak investigations conducted during a public health emergency. The processing of COVID‐19 data is necessary for reasons of public interest in the area of public health, such as protecting against serious cross‐border threats to health or ensuring high standards of quality and safety of health care; therefore, this study was exempted from institutional review board approval (Regulation EU 2016/679 GDPR). The school setting screening was performed upon informed consent of participants, pursuant the directive issued by Lombardy Region (Prot. G1.2021.0013306 02/03/2021).

## AUTHOR CONTRIBUTIONS

PP, MT, MA, and SM conceived the study. PP and MM wrote the first draft of the manuscript. MM wrote the code and performed the analyses. SD, GM, CA, SL, and MT collected data. MT and SD verified all data. GG, VdA, VM, AZ, and FT contributed to data interpretation. PP, SD, MT, MA, and SM supervised the study. All authors read, reviewed, and approved the final version of the manuscript.

### PEER REVIEW

The peer review history for this article is available at https://publons.com/publon/10.1111/irv.13049.

## Supporting information


**Table S1.** Result of the generalized linear model estimating the risk of infection after an exposure
**Figure S1.** Schematic representation of the sampling algorithm adopted to reconstruct the transmission chains using multiple exposures and all potential infection episodes identified during the epidemiological investigations.
**Figure S2. A)** Distribution of secondary infections generated by identified positive cases, as obtained using the entire set of exposure events identified during contact tracing operations. **B)** Transmission matrix representing the average number of infections caused in each age group by positive cases of different ages, as obtained using the entire set of exposure events identified during contact tracing operations.

## Data Availability

Aggregate data analyzed during this study will be included in this published article as the Supporting Information.
