# Supplementary material for: Estimating SARS‐CoV‐2 transmission in educational settings: A retrospective cohort study
Source: Influenza Other Respir Viruses. 2022 Sep 20;17(1):e13049. doi: 10.1111/irv.13049 (PMC9538978; doi:10.1111/irv.13049)
Supplement: Supplementary file 1 — Table S1. Result of the generalized linear model estimating the risk of infection after an exposure Figure S1. Schematic representation of the sampling algorithm adopted to reconstruct the transmission chains using multiple exposures and all potential infection episodes identified during the epidemiological investigations. Figure S2. A) Distribution of secondary infections generated by identified positive cases, as obtained using the entire set of exposure events identified during contact tracing operations. B) Transmission matrix representing the average number of infections caused in each age group by positive cases of different ages, as obtained using the entire set of exposure events identified during contact tracing operations. [file IRV-17-e13049-s001.docx]

**Appendix**

Estimating SARS-CoV-2 transmission in educational settings: a retrospective cohort study

Mattia Manica ^1,2,§^, Piero Poletti^1,2,§^, Silvia Deandrea^3,§^, Giansanto Mosconi^3,4^, Cinzia Ancarani^3^, Silvia Lodola^3^, Giorgio Guzzetta^1,2^, Valeria d'Andrea^1^, Valentina Marziano^1^, Agnese Zardini^1^, Filippo Trentini^1,5^, Anna Odone^4^, Marcello Tirani^6,#^, Marco Ajelli^7,#^, Stefano Merler^1,2,#,*^

^§^ equally contributed
^#^ joint senior authors

^*^ corresponding author*:* merler@fbk.eu

^1^ Center for Health Emergencies, Bruno Kessler Foundation, Trento, Italy

^2^ Epilab-JRU, FEM-FBK Joint Research Unit, Trento, Italy

^3^ Prevention Department, Agency for Health Protection, Pavia, Italy

^4^ Department of Public Health, Experimental and Forensic Medicine, University of Pavia, Pavia, Italy

^5^ Dondena Centre for Research on Social Dynamics and Public Policy, and CovidCrisisLab, Bocconi University, Milan, Italy

^6^ Directorate General for Health, Lombardy Region, Milan, Italy

^7^ Laboratory for Computational Epidemiology and Public Health, Department of Epidemiology and Biostatistics, Indiana University School of Public Health, Bloomington, IN, USA

***Risk of infection after exposure to SARS-CoV-2.***

Table S1 shows the results we obtained with a generalized linear mixed-effects model (GLMM) with logit link applied to PCR test results after exposure to a SARS-CoV-2 positive case.

**Table S1.** Result of the generalized linear model estimating the risk of infection after an exposure

| **Parameter** | **Estimate** | **SE** | **Z value** | **P values** |
| --- | --- | --- | --- | --- |
| Reference: Female contact, Female infector, exposure in Household | -0.630 | 0.195 | -3.23 | 0.001 |
| Age contact (std) | 0.255 | 0.114 | 2.23 | 0.026 |
| Sex contact (Male) | -0.302 | 0.195 | -1.55 | 0.122 |
| Age infector (std) | 0.044 | 0.107 | 0.41 | 0.682 |
| Sex infector (Male) | -0.345 | 0.202 | -1.71 | 0.088 |
| exposure at School | -0.186 | 0.291 | 0.64 | 0.523 |
| exposure in the Community | -0.307 | 0.231 | -1.33 | 0.184 |

Age of contact was standardized, mean = 30.35, standard deviation = 23.4

Age of infector was standardized, mean = 24.14, standard deviation = 21.39

***Additional results from the reconstruction of transmission chains***

From all available records, we identified a total of 976 potential exposure events. Information about the age, sex, household, and school of cases and their close contact was available for 726 exposures, involving 221 potential infectors and 627 contacts. Of these contacts, 261 tested positive and 366 tested negative.


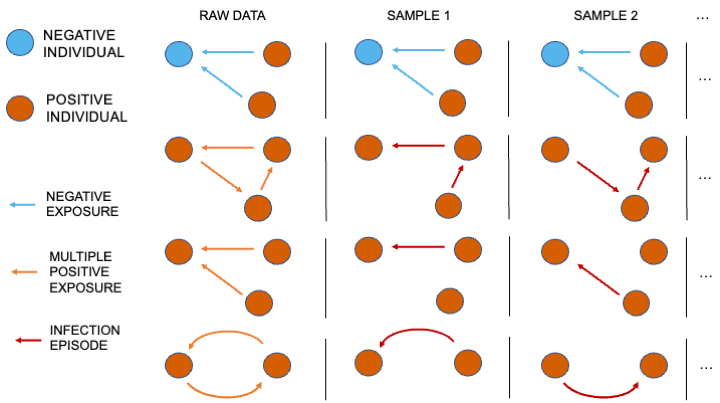


**Figure S1.** Schematic representation of the sampling algorithm adopted to reconstruct the transmission chains using multiple exposures and all potential infection episodes identified during the epidemiological investigations.

Based on these 726 exposures, we reconstructed the potential transmission chains occurred during the outbreak by resampling unclear exposures as described in the main text. We found that 154 (95%PI: 146-161) positive individuals (42.9%, 95%PI: 41.7-44.1% of the analyzed potential infectors) did not cause any secondary infection. A higher proportion of individuals causing onward transmission was found among positive students (48.8% vs 29.9%, on average). The average number of secondary infections caused by any positive individual was estimated to be 0.6 (95%PI: 0.59-0.61). Positive students caused on average 1.26 (95%PI: 1.18-1.33) secondary cases. No relevant differences were found in the number of secondary infections caused by school personnel and by individuals unrelated with the school setting (0.37 vs 0.43, on average).

We estimated the distribution of the number of secondary infections to follow a negative binomial distribution with overdispersion (shape parameter) 0.53 (95%PI: 0.47-0.61), implying that 20% of infectors were responsible for 75-80% of all secondary cases (Figure S2A). A similar heterogeneity in the transmission was found among students (overdispersion: 0.53, 95%PI: 0.45-0.62). We estimated that 118 (95%PI: 110-125) infection episodes were linked to a household contact, out of the 291 (95%PI: 283-298) estimated household exposures; 37 (95%PI: 36-40) infections were linked to a transmission in school (out of the 170, 95%PI: 168-172, estimated exposures); 59 (95%PI: 55-64) infections occurred in the community (185, 95%PI: 181-190, estimated exposures. Accordingly, infection episodes represented 40.5% (95%PI: 38.9-41.9%), 22.2% (95%PI: 21.4-23.3%), and 32% (95%PI: 30.4-33.7%) of all estimated exposures occurred in the household, school, and community, respectively. The average number of secondary cases caused by any positive individual at home and in the community was 0.33 (95%PI: 0.31-0.34) and 0.17 (95%PI: 0.15-0.18), respectively. Positive students caused an additional 0.5 (95%PI: 0.47-0.52) cases among school-related contacts (schoolmates or school personnel). Based on the identified infection episodes we reconstructed an age-specific matrix representing the average number of infections caused in each age group by a positive case, stratified by the age of the infector (Figure S2B).


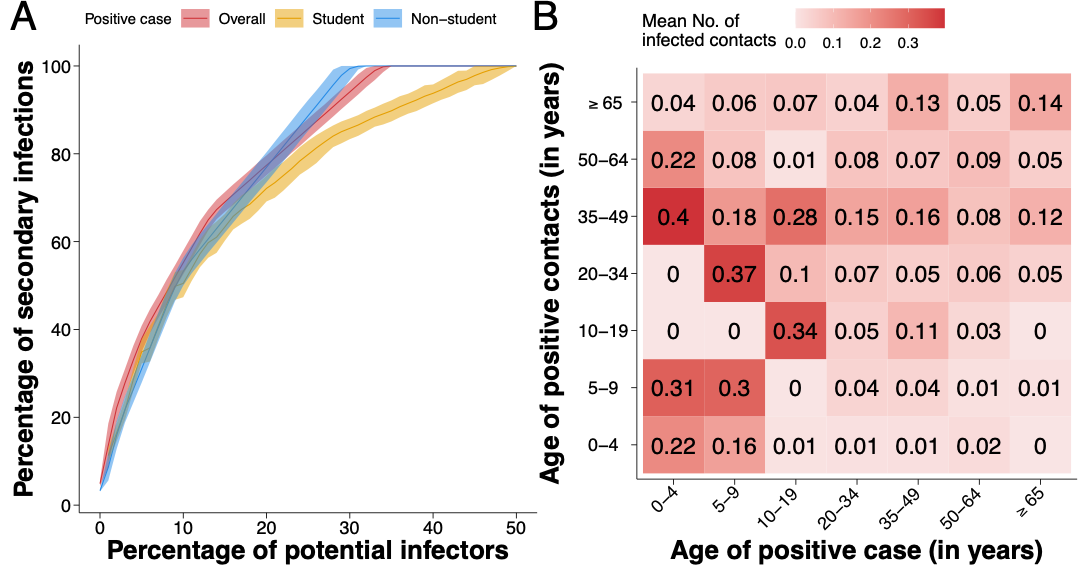


**Figure S2. A)** Distribution of secondary infections generated by identified positive cases, as obtained using the entire set of exposure events identified during contact tracing operations. **B)** Transmission matrix representing the average number of infections caused in each age group by positive cases of different ages, as obtained using the entire set of exposure events identified during contact tracing operations.
